# International Society of Sports Nutrition Position Stand: protein and exercise

**DOI:** 10.1186/s12970-017-0177-8

**Published:** 2017-06-20

**Authors:** Ralf Jäger, Chad M. Kerksick, Bill I. Campbell, Paul J. Cribb, Shawn D. Wells, Tim M. Skwiat, Martin Purpura, Tim N. Ziegenfuss, Arny A. Ferrando, Shawn M. Arent, Abbie E. Smith-Ryan, Jeffrey R. Stout, Paul J. Arciero, Michael J. Ormsbee, Lem W. Taylor, Colin D. Wilborn, Doug S. Kalman, Richard B. Kreider, Darryn S. Willoughby, Jay R. Hoffman, Jamie L. Krzykowski, Jose Antonio

**Affiliations:** 1 Increnovo LLC, Milwaukee, WI USA; 2 Exercise and Performance Nutrition Laboratory, School of Health Sciences, Lindenwood University, St. Charles, MO USA; 3 Performance & Physique Enhancement Laboratory, University of South Florida, Tampa, FL USA; 4 Metabolic Precision Certifications, Queensland, Australia; 5 BioTRUST Nutrition, Irving, TX USA; 6 The Center for Applied Health Sciences, Stow, OH USA; 7 Department of Geriatrics, University of Arkansas for Medical Sciences, Little Rock, AR USA; 8 IFNH Center for Health & Human Performance, Department of Kinesiology & Health, Rutgers, the State University of New Jersey, New Brunswick, New Jersey USA; 9 Applied Physiology Laboratory, Department of Exercise and Sport Science, University of North Carolina, Chapel Hill, NC USA; 10 Institute of Exercise Physiology and Wellness, University of Central Florida, Orlando, FL USA; 11 Human Nutrition and Metabolism Laboratory, Health and Exercise Sciences Department, Skidmore College, Saratoga Springs, NY 12866 USA; 12 Department of Nutrition, Food and Exercise Sciences, Institute of Sport Sciences and Medicine, Florida State University, Tallahassee, USA; 13 Biokinetics, Exercise and Leisure Studies, University of KwaZulu-Natal, Durban, 4000 South Africa; 14 Human Performance Laboratory, University of Mary Hardin-Baylor UMHB, Belton, TX 76513 USA; 15 Department of Nutrition & Endocrinology, QPS, Miami, FL USA; 16 Exercise & Sport Nutrition Lab, Human Clinical Research Facility, Department of Health & Kinesiology, Texas A&M University, College Station, TX USA; 17 Exercise and Biochemical Nutrition Laboratory, Department of Health, Human Performance, and Recreation, Baylor University, Waco, TX USA; 18 Department of Human Movement Sciences, Carroll University, Waukesha, WI USA; 19 Department of Health and Human Performance, Nova Southeastern University, Davie, FL USA

## Abstract

The International Society of Sports Nutrition (ISSN) provides an objective and critical review related to the intake of protein for healthy, exercising individuals. Based on the current available literature, the position of the Society is as follows:

1. An acute exercise stimulus, particularly resistance exercise, and protein ingestion both stimulate muscle protein synthesis (MPS) and are synergistic when protein consumption occurs before or after resistance exercise.
2. For building muscle mass and for maintaining muscle mass through a positive muscle protein balance, an overall daily protein intake in the range of 1.4–2.0 g protein/kg body weight/day (g/kg/d) is sufficient for most exercising individuals, a value that falls in line within the Acceptable Macronutrient Distribution Range published by the Institute of Medicine for protein.
3. Higher protein intakes (2.3–3.1 g/kg/d) may be needed to maximize the retention of lean body mass in resistance-trained subjects during hypocaloric periods.
4. There is novel evidence that suggests higher protein intakes (>3.0 g/kg/d) may have positive effects on body composition in resistance-trained individuals (i.e., promote loss of fat mass).
5. Recommendations regarding the optimal protein intake per serving for athletes to maximize MPS are mixed and are dependent upon age and recent resistance exercise stimuli. General recommendations are 0.25 g of a high-quality protein per kg of body weight, or an absolute dose of 20–40 g.
6. Acute protein doses should strive to contain 700–3000 mg of leucine and/or a higher relative leucine content, in addition to a balanced array of the essential amino acids (EAAs).
7. These protein doses should ideally be evenly distributed, every 3–4 h, across the day.
8. The optimal time period during which to ingest protein is likely a matter of individual tolerance, since benefits are derived from pre- or post-workout ingestion; however, the anabolic effect of exercise is long-lasting (at least 24 h), but likely diminishes with increasing time post-exercise.
9. While it is possible for physically active individuals to obtain their daily protein requirements through the consumption of whole foods, supplementation is a practical way of ensuring intake of adequate protein quality and quantity, while minimizing caloric intake, particularly for athletes who typically complete high volumes of training.
10. Rapidly digested proteins that contain high proportions of essential amino acids (EAAs) and adequate leucine, are most effective in stimulating MPS.
11. Different types and quality of protein can affect amino acid bioavailability following protein supplementation.
12. Athletes should consider focusing on whole food sources of protein that contain all of the EAAs (i.e., it is the EAAs that are required to stimulate MPS).
13. Endurance athletes should focus on achieving adequate carbohydrate intake to promote optimal performance; the addition of protein may help to offset muscle damage and promote recovery.
14. Pre-sleep casein protein intake (30–40 g) provides increases in overnight MPS and metabolic rate without influencing lipolysis.

## Background

In 2007, the International Society of Sports Nutrition (ISSN) published its first position stand devoted to the science and application of dietary protein intake [1]. Subsequently, this paper has been accessed more than 200,000 times and continues to serve as a key reference on the topic. In the past ten years, there have been continued efforts to advance the science and application of dietary protein intake for the benefit of athletes and fitness-minded individuals. This updated position stand includes new information and addresses the most important dietary protein categories that affect physically active individuals across domains such as exercise performance, body composition, protein timing, recommended intakes, protein sources and quality, and the preparation methods of various proteins.

## Benefits on exercise performance

Most of the scientific research investigating the effects of protein intake on exercise performance has focused on supplemental protein intake. From a broad perspective, the dependent measures of these studies can be categorized into two domains:

- Endurance exercise performance
- Resistance exercise performance (increases in maximal strength)

### Endurance exercise performance

Very few studies have investigated the effects of prolonged periods (one week or more) of dietary protein manipulation on endurance performance. Macdermid and colleagues [2] compared the influence of an isoenergetic, high-protein/moderate-carbohydrate diet (3.3 and 5.9 g of protein and carbohydrate/kg body weight per day, respectively) with a diet that was more typical of an endurance athlete (1.3 and 7.9 g of protein and carbohydrate/kg body weight per day, respectively) in endurance-trained cyclists. The trained cyclists ingested each diet for a 7-day period in a randomized, crossover fashion. Before and following the 7-day diet intervention, a self-paced cycling endurance time trial was conducted as the primary measure of exercise performance. At the end of the treatment period, it took cyclists on the higher protein diet 20% more time to complete the self-paced time trial - significantly longer than for those on the lower protein/higher carbohydrate diet. This finding is not surprising given that dietary protein is not a preferred energy source and the dietary carbohydrate intakes in the higher protein treatment were below recommended intakes for endurance athletes (6–10 g of carbohydrate/kg/d) [3]. It should be noted however that a 7-day treatment period is exceedingly brief. It is unknown what the effect of a higher protein diet would be over the course of several weeks or months.

In another study [4] utilizing highly trained cyclists during a period of increased training intensity, it was observed that 3 g of protein/kg/d offered no improvements in a simulated time trial as compared to 1.5 g of protein/kg body weight/day. Carbohydrate intake was kept constant (6 g/kg/d) in both the moderate and high protein treatments during this three-week intervention. Although the number of investigations is limited, it appears as if increasing protein intakes above recommended intakes does not enhance endurance performance [2, 4, 5].

In addition to these studies that spanned one to three weeks, several acute-response (single feeding and exercise sessions) studies exist, during which protein was added to a carbohydrate beverage prior to or during endurance exercise. Similarly, most of these interventions also reported no added improvements in endurance performance when protein was added to a carbohydrate beverage as compared to carbohydrate alone [6–9]. An important research design note, however, is that those studies which reported improvements in endurance performance when protein was added to a carbohydrate beverage before and during exercise all used a time-to-exhaustion test [10–12]. When specifically interested in performance outcomes, a time trial is preferred as it better mimics competition and pacing demands.

In conclusion, added protein does not appear to improve endurance performance when given for several days, weeks, or immediately prior to and during endurance exercise. While no ergogenic outcomes may be evident, the scientific literature is consistent in reporting that adding protein to a carbohydrate beverage/gel during exhaustive endurance exercise suppresses markers of muscle damage (creatine kinase) 12 to 24 h post-exercise [8, 11–13] and decreases the endurance athletes’ feelings of muscular soreness [6–8, 13]. For these reasons, it seems prudent to recommend for endurance athletes to ingest approximately 0.25 g of protein/kg body weight per hour of endurance exercise (in addition to the athlete’s regular carbohydrate intake) to suppress markers of muscle damage and improve subjective feelings of muscular soreness [11, 12]. Another important consideration relates to the impact of ingesting protein along with carbohydrate on rates of protein synthesis and balance during prolonged bouts of endurance exercise. Beelen and colleagues [14] determined that adding protein to carbohydrate consumption throughout a prolonged bout of endurance exercise promotes a higher whole body net protein balance, but the added protein does not exert any further impact on rates of MPS. While performance outcomes were not measured, these results shift the focus of nutrient ingestion during prolonged bouts of endurance exercise to the ingestion of carbohydrate.

#### Key points

- When adequate carbohydrate is delivered, adding protein to carbohydrate does not appear to improve endurance performance over the course of a few days or weeks.
- Adding protein during or after an intensive bout of endurance exercise may suppress the rise in plasma proteins linked to myofibrillar damage and reduce feelings of muscle soreness.
- There are relatively few investigations on the effects of protein supplementation on endurance performance.

### Resistance exercise performance

The extent to which protein supplementation, in conjunction with resistance training, enhances maximal strength is contingent upon many factors, including:

- Resistance-training program variables (such as intensity, volume, and progression)
- Length of the resistance-training program/intervention
- Training status of the participants engaging in the resistance-training program
- Energy intake in the diet
- Quality and quantity of protein intake (with an emphasis on leucine content of the protein)
- Co-ingestion of additional dietary ingredients that may favorably impact strength (e.g. creatine, HMB)

Taking each of these variables into consideration, the effects of supplemental protein consumption has on maximal strength enhancement are varied, with a majority of the investigations reporting no benefit [15–25] and a few reporting improvements in maximal strength [26–29]. With limited exceptions [16, 18, 23, 27], most of the studies utilized young, healthy, untrained males as participants. In one investigation examining college football athletes supplementing with a proprietary milk protein supplement (two servings of 42 g per day) for 12 weeks, a 14.5% increase in maximal squat strength was observed compared to a 6.9% increase in the placebo group [28]. These differences were statistically significant. When females were the only sex investigated, the outcomes consistently indicated that supplemental protein does not appear to enhance maximal strength at magnitudes that reach statistical significance. Hida et al. [30] reported that females supplementing with 15 g of egg white protein (which raised daily protein intake to 1.23 g of protein/kg body weight/day) experienced no improvements in maximal upper and lower body strength as compared to a carbohydrate placebo (ingesting one gram of protein/kg body weight/day) over an 8-week period. An important note for this study is that 15 g of egg protein is considered by many to be a sub-optimal dose [31]. However, others have advocated that the total daily intake of protein might be as important or more important [32]. In another study, Josse et al. [33] reported that non-resistance trained females supplementing with one liter of skimmed bovine milk (providing 36 g of protein) after resistance exercise improved maximal strength in seven of nine measures as compared to a carbohydrate placebo group, but only the improvements to maximal bench press strength attained statistical significance compared to the placebo. In contrast, Taylor and colleagues [34] reported that pre- and post-exercise whey protein ingestion significantly increased maximal upper-body strength (+4.9 kg bench press one repetition maximum) in comparison to changes seen when a maltodextrin placebo (+2.3 kg) was ingested in a group of female collegiate basketball players over an 8-week period.

In summary, while research investigating the addition of supplemental protein to a diet with adequate energy and nutrient intakes is inconclusive in regards to stimulating strength gains in conjunction with a resistance-training program to a statistically significant degree, greater protein intakes that are achieved from both dietary and supplemental sources do appear to have some advantage. Hoffman and colleagues [29] reported that in athletes consuming daily protein intakes above 2.0 g/kg/d which included protein intakes from both diet and supplements, a 22% and 42% increase in strength was noted in both the squat and bench press exercises during off-season conditioning in college football players compared to athletes that consumed only the recommended levels (1.6–1.8 g/kg/d) for strength/power athletes. Further, it is important to highlight that in most studies cited, protein intervention resulted in greater but non-statistically significant strength improvements as compared to the placebo/control condition. Cermak and colleagues [35] pooled the outcomes from 22 separate clinical trials to yield 680 subjects in their statistical analysis and found that protein supplementation with resistance training resulted in a 13.5 kg increase (95% Confidence Interval: 6.4–20.7 kg) in lower-body strength when compared to changes seen when a placebo was provided. A similar conclusion was also drawn by Pasiakos et al. [36] in a meta-analysis where they reported that in untrained participants, protein supplementation might exert very little benefit on strength during the initial weeks of a resistance training program, but as duration, frequency and volume of resistance training increased, protein supplementation may favorably impact skeletal muscle hypertrophy and strength.

#### Key points:

- Results from many single investigations indicate that in both men and women protein supplementation exerts a small to modest impact on strength development.
- Pooled results of multiple studies using meta-analytic and other systematic approaches consistently indicate that protein supplementation (15 to 25 g over 4 to 21 weeks) exerts a positive impact on performance.

## Body composition

Improving one’s body composition through the loss of fat mass and increasing fat-free mass is often associated with improvements in physical performance. In this respect, many published investigations report that protein supplementation results in significant improvements in lean body weight/cross-sectional areas as compared to placebo treatments [15, 17, 21–23, 26, 27, 33, 37]. Andersen et al. [15] examined 22 healthy men that completed a 14-week resistance-training program (3 days/week consisting of 3–4 sets of lower body exercises) while supplementing with either 25 g of a high-quality protein blend or 25 g of carbohydrate. When the blend of milk proteins was provided, significantly greater increases in fat-free mass, muscle cross-sectional area in both the Type I and Type II muscle fibers occurred when compared to changes seen with carbohydrate consumption. Collectively, a meta-analysis by Cermak and colleagues [35] reported a mean increase in fat-free mass of 0.69 kg (95% Confidence Interval: 0.47–0.91 kg) when protein supplementation was provided versus a placebo during a resistance-training program. Other reviews by Tipton, Phillips and Pasiakos, respectively, [36, 38, 39] provide further support that protein supplementation (15–25 g over 4–14 weeks) augments lean mass accretion when combined with completion of a resistance training program.

Beyond accretion of fat-free mass, increasing daily protein intake through a combination of food and supplementation to levels above the recommended daily allowance (RDA) (RDA 0.8 g/kg/day, increasing to 1.2–2.4 g/kg/day for the endurance and strength/power athletes) while restricting energy intake (30–40% reduction in energy intake) has been demonstrated to maximize the loss of fat tissue while also promoting the maintenance of fat-free mass [40–45]. The majority of this work has been conducted using overweight and obese individuals who were prescribed an energy-restricted diet that delivered a greater ratio of protein relative to carbohydrate. As a classic example, Layman and investigators [40] randomized obese women to consume one of two restricted energy diets (1600–1700 kcals/day) that were either higher in carbohydrates (>3.5: carbohydrate-to-protein ratio) or protein (<1.5: carbohydrate-to-protein ratio). Groups were further divided into those that followed a five-day per week exercise program (walking + resistance training, 20–50 min/workout) and a control group that performed light walking of less than 100 min per week. Greater amounts of fat were lost when higher amounts of protein were ingested, but even greater amounts of fat loss occurred when the exercise program was added to the high-protein diet group, resulting in significant decreases in body fat. Using an active population that ranged from normal weight to overweight (BMI: 22–29 kg/m^2^), Pasiakos and colleagues [42] examined the impact of progressively increasing dietary protein over a 21-day study period. An aggressive energy reduction model was employed that resulted in each participant reducing their caloric intake by 30% and increasing their energy expenditure by 10%. Each person was randomly assigned to consume a diet that contained either 1× (0.8 g/kg), 2× (1.6 g/kg) or 3× (2.4 g/kg) the RDA for protein. Participants were measured for changes in body weight and body composition. While the greatest body weight loss occurred in the 1× RDA group, this group also lost the highest percentage of fat-free mass and lowest percentage of fat mass. The 2× and 3× RDA groups lost significant amounts of body weight that consisted of 70% and 64% fat mass, respectively.

Collectively, these results indicate that increasing dietary protein can promote favorable adaptations in body composition through the promotion of fat-free mass accretion when combined with a hyperenergetic diet and a heavy resistance training program and can also promote the loss of fat mass when higher intakes of daily protein (2-3× the RDA) are combined with an exercise program and a hypoenergetic diet.

### Key points:

- When combined with a hyperenergetic diet and a heavy resistance-training program, protein supplementation may promote increases in skeletal muscle cross-sectional area and lean body mass.
- When combined with a resistance-training program and a hypoenergetic diet, an elevated daily intake of protein (2 – 3× the RDA) can promote greater losses of fat mass and greater overall improvements in body composition.

## Protein timing

Thanks to seminal work by pioneering research groups [37, 46, 47], by the 1990’s it was clear that exercise and macronutrient consumption interact synergistically to provide a net anabolic effect far greater than either feeding or exercise alone. In the absence of feeding, muscle protein balance remains negative in response to an acute bout of resistance exercise [48]. Tipton et al. [49] were one of the first groups to illustrate that an acute feeding of amino acids significantly increases rates of muscle protein synthesis (MPS). Later, Burd et al. [50] indicated that the combination of acute, exhaustive resistance exercise increases the muscle’s anabolic responsiveness to whey protein provision for up to 24 h. In addition to heightened anabolic sensitivity that stems from the combination of resistance exercise and protein/amino acid feeding, the importance of the EAAs with respect to muscle protein growth has also been elucidated. Tipton et al. [51] first indicated that nonessential amino acids were not necessary to stimulate MPS. Subsequently, these conclusions were supported by Borsheim [52] and Volpi [53]. The study by Borsheim also documented a dose-response outcome characterized by a near doubling of net protein balance in response to a three to six gram dose of the EAAs [52]. Building on this work, Tipton et al. [54] reported that EAAs (9–15 g dose) before and after resistance exercise promoted higher net protein accretion, not just 3 or 4 h post exercise but also over a 24-h period [55]. These findings formed the theoretical concept of protein timing for resistance exercise that has since been transferred to not only other short-duration, high-intensity activities [56] but also endurance-based sports [57] and subsequent performance outcomes [58]. The strategic consumption of nutrition, namely protein or various forms of amino acids, in the hours immediately before and during exercise (i.e., peri-workout nutrition) has been shown to maximize muscle repair and optimize strength- and hypertrophy-related adaptations [59, 60]. While earlier investigations reported positive effects from consumption of amino acids [37, 46, 61], it is now clear that intact protein supplements such as egg, whey, casein, beef, soy and even whole milk can evoke an anabolic response that can be similar or greater in magnitude to free form amino acids, assuming ingestion of equal EAA amounts [62–64].

For instance, whey protein ingested close to resistance exercise, promotes a higher activation (phosphorylation) of mTOR (a key signaling protein found in myocytes that is linked to the synthesis of muscle proteins) and its downstream mRNA translational signaling proteins (i.e., p70s6 kinase and eIF4BP) that further suggests timed ingestion of protein may favorably promote heightened muscle hypertrophy [21, 62]. Moreover, it was found that the increased mTOR signaling corresponded with significantly greater muscle hypertrophy after 10 weeks of training [65]. However, the hypertrophic differences between protein consumption and a non-caloric placebo appeared to plateau by week 21, despite a persistently greater activation of this molecular signaling pathway from supplementation. Results from other research groups [56–58, 66] show that timing of protein near (± 2 h) aerobic and anaerobic exercise training appears to provide a greater activation of the molecular signalling pathways that regulate myofibrillar and mitochondrial protein synthesis as well as glycogen synthesis.

It is widely reported that protein consumption directly after resistance exercise is an effective way to acutely promote a positive muscle protein balance [31, 55, 67], which if repeated over time should translate into a net gain or hypertrophy of muscle [68]. Pennings and colleagues [69] reported an increase in both the delivery and incorporation of dietary proteins into the skeletal muscle of young and older adults when protein was ingested shortly after completion of exercise. These findings and others add to the theoretical basis for consumption of post-protein sooner rather than later after exercise, since post workout MPS rates peak within three hours and remain elevated for an additional 24–72 h [50, 70]. This extended time frame also provides a rationale for both immediate and sustained (i.e., every 3–4 h) feedings to optimize impact. These temporal considerations would also capture the peak elevation in signalling proteins shown to be pivotal for increasing the initiation of translation of muscle proteins, which for the most part appears to peak between 30 and 60 min after exercise [71]. Finally, while some investigations have shown that a rapid increase in amino acids (aminoacidemia) from a protein dose immediately after or surrounding exercise stimulates increased adaptations to resistance training [72, 73], others examining competitive strength/power athletes reported no advantage from pre/post supplement feedings compared to similar feedings in morning and evening hours [74]. However, these differences may be related to the type of protein used between the studies. The studies showing positive effects of protein timing used milk proteins, whereas the latter study used a collagen based protein supplement.

While a great deal of work has focused on post-exercise protein ingestion, other studies have suggested that pre-exercise and even intra-exercise ingestion may also support favorable changes in MPS and muscle protein breakdown [14, 54, 75–78]. Initially, Tipton and colleagues [54] directly compared immediate pre-exercise and immediate post-exercise ingestion of a mixture of carbohydrate (35 g) and EAAs (6 g) combination on changes in MPS. They reported that pre-exercise ingestion promoted higher rates of MPS while also demonstrating that nutrient ingestion prior to exercise increased nutrient delivery to a much greater extent than other (immediate or one hour post-exercise) time points. These results were later challenged by Fujita in 2009 who employed an identical study design with a different tracer incorporation approach and concluded there was no difference between pre- or post-exercise ingestion [75]. Subsequent work by Tipton [79] also found that similar elevated rates of MPS were achieved when ingesting 20 g of a whey protein isolate immediately before or immediately after resistance exercise.

At this point, whether any particular time of protein ingestion confers any unique advantage over other time points throughout a 24-h day to improve strength and hypertrophy has yet to be adequately investigated. To date, although a substantial amount of literature discusses this concept [60, 80], a limited number of training studies have assessed whether immediate pre- and post-exercise protein consumption provides unique advantages compared to other time points [72, 73, 81]. Each study differed in population, training program, environment and nutrition utilized, with each reporting a different result. What is becoming clear is that the subject population, nutrition habits, dosing protocols on both training and non-training days, energy and macronutrient intake, as well as the exercise bout or training program itself should be carefully considered alongside the results. In particular, the daily amount of protein intake seems to operate as a key consideration because the benefits of protein timing in relation to the peri-workout period seem to be lessened for people who are already ingesting appropriate amounts of protein (e.g. ≥1.6 g/kg/day). This observation can be seen when comparing the initial results of Cribb [72], Hoffman [74] and most recently with Schoenfeld [82]; however, one must also consider that the participants in the Hoffman study may have been hypocaloric as they reported consuming approximately 30 kcal/kg in all groups across the entire study. A literature review by Aragon and Schoenfeld [83] determined that while compelling evidence exists showing muscle is sensitized to protein ingestion following training, the increased sensitivity to protein ingestion might be greatest in the first five to six hours following exercise. Thus, the importance of timing may be largely dependent on when a pre-workout meal was consumed, the size and composition of that meal and the total daily protein in the diet. In this respect, a pre-exercise meal will provide amino acids during and after exercise and therefore it stands to reason there is less need for immediate post-exercise protein ingestion if a pre-exercise meal is consumed less than five hours before the anticipated completion of a workout. A meta-analysis by Schoenfeld et al. [84] found that consuming protein within one-hour post resistance exercise had a small but significant effect on increasing muscle hypertrophy compared to delaying consumption by at least two hours. However, sub-analysis of these results revealed the effect all but disappeared after controlling for the total intake of protein, indicating that favorable effects were due to unequal protein intake between the experimental and control groups (∼1.7 g/kg versus 1.3 g/kg, respectively) as opposed to temporal aspects of feeding. The authors concluded that total protein intake was the strongest predictor of muscular hypertrophy and that protein timing likely influences hypertrophy to a lesser degree. However, the conclusions from this meta-analysis may be questioned because the majority of the studies analyzed were not protein timing studies but rather protein supplementation studies. In that respect, the meta-analysis provides evidence that protein supplementation (i.e., greater total daily protein intake) may indeed confer an anabolic effect. While a strong rationale remains to support the concept that the hours immediately before or after resistance exercise represents an opportune time to deliver key nutrients that will drive the accretion of fat-free mass and possibly other favorable adaptations, the majority of available literature suggests that other factors may indeed be operating to a similar degree that ultimately impact the observed adaptations. In this respect, a key variable that must be accounted for is the absolute need for energy and protein required to appropriately set the body up to accumulate fat-free mass.

A review by Bosse and Dixon [84] critically summarized the available literature on protein supplementation during resistance exercise and hypothesized that protein intake may need to increase by as much as 59% above baseline levels for significant changes in fat-free mass to occur. Finally, it should be noted that for many athletes, consuming a post- or pre-workout protein-containing meal represents a feeding opportunity with little downside, since there is no benefit from not consuming protein pre- and/or post-exercise. In other words, not consuming protein-containing foods/supplements post-exercise is a strategy that provides no benefit whatsoever. Thus, the most practical recommendation is to have athletes consume a meal during the post-workout (or pre-workout) time period since it may either help or have a neutral effect.

In younger subjects, the ingestion of 20–30 g of any high biological value protein before or after resistance exercise appears to be sufficient to maximally stimulate MPS [21, 64]. More recently, Macnaughton and colleagues [85] reported that 40 g of whey protein ingestion significantly increased the MPS responses compared to a 20 g feeding after an acute bout of whole-body resistance exercise, and that the absolute protein dose may operate as a more important consideration than providing a protein dose that is normalized to lean mass. Free form EAAs, soy, milk, whey, caseinate, and other protein hydrolysates are all capable of activating MPS [86]. However, maximal stimulation of MPS, which results in higher net muscle protein accretion, is the product of the total amount of EAA in circulation as well as the pattern and appearance rate of aminoacidemia that modulates the MPS response [86]. Recent work has clarified that whey protein provides a distinct advantage over other protein sources including soy (considered another fast absorbing protein) and casein (a slower acting protein source) on acute stimulation of MPS [86, 87]. Importantly, an elegant study by West and investigators [87] sought to match the delivery of EAAs in feeding patterns that replicated how whey and casein are digested. The authors reported that a 25 g dose of whey protein that promoted rapid aminoacidemia further enhanced MPS and anabolic signaling when compared to an identical total dose of whey protein when delivered as ten separate 2.5 g doses intended to replicate a slower digesting protein. The advantages of whey protein are important to consider, particularly as all three sources rank similarly in assessments of protein quality [88]. In addition to soy, other plant sources (e.g., pea, rice, hemp, etc.) have garnered interest as potential protein sources to consider. Unfortunately, research that examines the ability of these protein sources to modulate exercise performance and training adaptations is limited at this time. One study conducted by Joy and investigators [89] compared the effect of supplementing a high-dose (48 g/day) of whey or rice protein in experienced resistance-trained subjects during an 8-week resistance training program. The investigators concluded that gains in strength, muscle thickness and body composition were similar between the two protein groups, suggesting that rice protein may be a suitable alternative to whey protein at promoting resistance training adaptations. Furthermore, differences in absorption kinetics, and the subsequent impact on muscle protein metabolism appear to extend beyond the degree of hydrolysis and amino acid profiles [69, 86, 90–92]. For instance, unlike soy more of the EAAs from whey proteins (hydrolysates and isolates) survive splanchnic uptake and travel to the periphery to activate a higher net gain in muscle [86]. Whey proteins (hydrolysates and isolates) appear to be the most extensively researched for pre/post resistance exercise supplementation, possibly because of their higher EAA and leucine content [93, 94], solubility, and optimal digestion kinetics [69]. These characteristics yield a high concentration of amino acids in the blood (aminoacidemia) [69, 87] that facilitates greater activation of MPS and net muscle protein accretion, in direct comparison to other protein choices [50, 69, 91]. The addition of creatine to whey protein supplementation appears to further augment these adaptations [27, 72, 95]; however, an optimal timing strategy for this combination remains unclear.

The timing of protein-rich meals consumed throughout a day has the potential to influence adaptations to exercise. Using similar methods, other studies over recent decades [53, 62, 87, 91, 96–100] have established the following:

- MPS increases approximately 30–100% in response to a protein-containing meal to promote a positive net protein balance, and the major contributing factor to this response is the EAA content.
- The anabolic response to feeding is pronounced but transient. During the post-prandial phase (1–4 h after a meal) MPS is elevated, resulting in a positive muscle protein balance. In contrast, MPS rates are lower in a fasted state and muscle protein balance is negative. Protein accretion only occurs in the fed state. The concentration of EAA in the blood (plasma) regulates protein synthesis rates within muscle at rest and post exercise. More recent work has established that protein-carbohydrate supplementation after strenuous endurance exercise stimulates contractile MPS via similar signaling pathways as resistance exercise [56, 57]. Most importantly, and as mentioned initially in this section, muscle appears to be “sensitized” to protein feeding for at least 24 h after exercise [50]. That is, the consumption of a protein-containing meal up to 24 h after a single bout of resistance exercise results in a higher net stimulation of MPS and protein accretion than the same meal consumed after 24 h of inactivity [50].
- The effect of insulin on MPS is dependent on its ability to increase amino acid availability, which does not occur when insulin is systematically increased (e.g., following feeding) [101]. In particular, insulin’s impact on net protein balance seems to operate most powerfully in an anti-catabolic manner on muscle [102]. However, insulin-mediated effects that reduce muscle protein breakdown peaks at low to moderate levels of insulin (~15–30 μIU/mL) [103, 104] that can be achieved by consumption of a 45-g dose of whey protein isolate alone [105]. Taken together, these results seem to indicate that post-workout carbohydrate supplementation offers very little contribution from a muscle development standpoint provided adequate protein is consumed. For example, Staples and colleagues [106] compared the impact of a carbohydrate + protein combination on rates of MPS and reported no further increases in MPS beyond what was seen with protein ingestion alone. Importantly, these results are not to be interpreted to mean that carbohydrate administration offers no potential effect for an athlete engaging in moderate to high volumes of training, but rather that benefits derived from carbohydrate administration appear to more favorably impact aspects of muscle glycogen recovery as opposed to stimulating muscle protein accretion.

### Pre-sleep protein intake

Eating before sleep has long been controversial [107–109]. However, a methodological consideration in the original studies such as the population used, time of feeding, and size of the pre-sleep meal confounds firm conclusions about benefits or drawbacks. Recent work using protein-rich beverages 30-min prior to sleep and two hours after the last meal (dinner) have identified pre-sleep protein consumption/ingestion as advantageous to MPS, muscle recovery, and overall metabolism in both acute and long-term studies [110, 111]. Results from several investigations indicate that 30–40 g of casein protein ingested 30-min prior to sleep [112] or via nasogastric tubing [113] increased overnight MPS in both young and old men, respectively. Likewise, in an acute setting, 30 g of whey protein, 30 g of casein protein, and 33 g of carbohydrate consumed 30-min prior to sleep resulted in an elevated morning resting metabolic rate in young fit men compared to a non-caloric placebo [114]. Similarly, although not statistically significant, morning increases in resting metabolic rate were reported in young overweight and/or obese women [115]. Interestingly, Madzima et al. [114] reported that subjects’ respiratory quotient measured during the morning after pre-sleep nutrient intake was unchanged only for the placebo and casein protein trials, while both carbohydrate and whey protein were increased compared to placebo. This infers that casein protein consumed pre-sleep maintains overnight lipolysis and fat oxidation. This finding was further supported by Kinsey et al. [116] using a microdialysis technique to measure interstitial glycerol concentrations overnight from the subcutaneous abdominal adipose tissue, reporting greater fat oxidation following consumption of 30 g of casein compared to a flavor and sensory-matched noncaloric placebo in obese men. Similar to Madzima et al. [114], Kinsey et al. [116] concluded that pre-sleep casein did not blunt overnight lipolysis or fat oxidation. Interestingly, the pre-sleep protein and carbohydrate ingestion resulted in elevated insulin concentrations the next morning and decreased hunger in this overweight population. Of note, it appears that exercise training completely ameliorates any rise in insulin when eating at night before sleep [117], while the combination of pre-sleep protein and exercise has been shown to reduce blood pressure and arterial stiffness in young obese women with prehypertension and hypertension [118]. In athletes, evening chocolate milk consumption has also been shown to influence carbohydrate metabolism in the morning, but not running performance [108]. In addition, data supports that exercise performed in the evening augments the overnight MPS response in both younger and older men [119–121].

To date, only a few studies involving nighttime protein ingestion have been carried out for longer than four weeks. Snijders et al. [122] randomly assigned young men (average age of 22 years) to consume a protein-centric supplement (27.5 g of casein protein, 15 g of carbohydrate, and 0.1 g of fat) or a noncaloric placebo every night before sleep while also completing a 12-week progressive resistance exercise training program (3 times per week). The group receiving the protein-centric supplement each night before sleep had greater improvements in muscle mass and strength over the 12-week study. Of note, this study was non-nitrogen balanced and the protein group received approximately 1.9 g/kg/day of protein compared to 1.3 g/kg/day in the placebo group. More recently, in a study in which total protein intake was equal, Antonio et al. [123] studied young healthy men and women that supplemented with casein protein (54 g) for 8 weeks either in the morning (any time before 12 pm) or the evening supplementation (90 min or less prior to sleep). They examined the effects on body composition and performance [123]. All subjects maintained their usual exercise program. The authors reported no differences in body composition or performance between the morning and evening casein supplementation groups. However, it is worth noting that, although not statistically significant, the morning group added 0.4 kg of fat free mass while the evening protein group added 1.2 kg of fat free mass, even though the habitual diet of the trained subjects in this study consumed 1.7 to 1.9 g/kg/day of protein. Although this finding was not statistically significant, it supports data from Burk et al. [81] indicating that casein-based protein consumed in the morning (10 am) and evening (10:30 pm) was more beneficial for increasing fat-free mass than consuming the protein supplement in the morning (10 am) and afternoon (~3:50 pm). It should be noted that the subjects in the Burk et al. study were resistance training. A retrospective epidemiological study by Buckner et al. [124] using NHANES data (1999–2002) showed that participants consuming 20, 25, or 30 g of protein in the evening had greater leg lean mass compared to subjects consuming protein in the afternoon. Thus, it appears that protein consumption in the evening before sleep might be an underutilized time to take advantage of a protein feeding opportunity that can potentially improve body composition and performance.

### Protein ingestion and meal timing

In addition to direct assessments of timed administration of nutrients, other studies have explored questions that center upon the pattern of when certain protein-containing meals are consumed. Paddon-Jones et al. [97] reported a correlation between acute stimulation of MPS via protein consumption and chronic changes in muscle mass. In this study, participants were given an EAA supplement three times a day for 28 days. Results indicated that acute stimulation of MPS provided by the supplement on day 1 resulted in a net gain of ~7.5 g of muscle over a 24-h period [97]. When extrapolated over the entire 28-day study, the predicted change in muscle mass corresponded to the actual change in muscle mass (~210 g) measured by dual-energy x-ray absorptiometry (DEXA) [97]. While these findings are important, it is vital to highlight that this study incorporated a bed rest model with no acute exercise stimulus while other work by Mitchell et al. [125] reported a lack of correlation between measures of acute MPS and the accretion of skeletal muscle mass.

Interestingly, supplementation with 15 g of EAAs and 30 g of carbohydrate produced a greater anabolic effect (increase in net phenylalanine balance) than the ingestion of a mixed macronutrient meal, despite the fact that both interventions contained a similar dose of EAAs [96]. Most importantly, the consumption of the supplement did not interfere with the normal anabolic response to the meal consumed three hours later [96]. The results of these investigations suggest that protein supplement timing between the regular “three square meals a day” may provide an additive effect on net protein accretion due to a more frequent stimulation of MPS. Areta et al. [126] were the first to examine the anabolic response in human skeletal muscle to various protein feeding strategies for a day after a single bout of resistance exercise. The researchers compared the anabolic responses of three different patterns of ingestion (a total of 80 g of protein) throughout a 12-h recovery period after resistance exercise. Using a group of healthy young adult males, the protein feeding strategies consisted of small pulsed (8 × 10 g), intermediate (4 × 20 g), or bolus (2 × 40 g) administration of whey protein over the 12-h measurement window. Results showed that the intermediate dosing (4 × 20 g) was superior for stimulating MPS for the 12-h experimental period. Specifically, the rates of myofibrillar protein synthesis were optimized throughout the day of recovery by the consumption of 20 g protein every three hours compared to large (2 × 40 g), less frequent servings or smaller but more frequent (8 × 10 g) patterns of protein intake [67]. Previously, the effect of various protein feeding strategies on skeletal MPS during an entire day was unknown. This study provided novel information demonstrating that the regulation of MPS can be modulated by the timing and distribution of protein over 12 h after a single bout of resistance exercise. However, it should be noted that an 80 g dose of protein over a 12-h period is quite low.

The logical next step for researchers is to extend these findings into longitudinal training studies to see if these patterns can significantly affect resistance-training adaptations. Indeed, published studies by Arnal [127] and Tinsley [128] have all made some attempt to examine the impact of adjusting the pattern of protein consumption across the day in combination with various forms of exercise. Collective results from these studies are mixed. Thus, future studies in young adults should be designed to compare a balanced vs. skewed distribution pattern of daily protein intake on the daytime stimulation of MPS (under resting and post-exercise conditions) and training-induced changes in muscle mass, while taking into consideration the established optimal dose of protein contained in a single serving for young adults. Without more conclusive evidence spanning several weeks, it seems pragmatic to recommend the consumption of at least 20-25 g of protein (~0.25 g/kg/meal) with each main meal with no more than 3–4 h between meals [126].

#### Key points

- In the absence of feeding and in response to resistance exercise, muscle protein balance remains negative.
- Skeletal muscle is sensitized to the effects of protein and amino acids for up to 24 h after completion of a bout of resistance exercise.
- A protein dose of 20–40 g of protein (10–12 g of EAAs, 1–3 g of leucine) stimulates MPS, which can help to promote a positive nitrogen balance.
- The EAAs are critically needed for achieving maximal rates of MPS making high-quality, protein sources that are rich in EAAs and leucine the preferred sources of protein.
- Studies have suggested that pre-exercise feedings of amino acids in combination with carbohydrate can achieve maximal rates of MPS, but protein and amino acid feedings during this time are not clearly documented to increase exercise performance.
- Ingestion of carbohydrate + protein or EAAs during endurance and resistance exercise can help to maintain a favorable anabolic hormone profile, minimize increases in muscle damage, promote increases in muscle cross-sectional area, and increase time to exhaustion during prolonged running and cycling.
- Post-exercise administration of protein when combined with suboptimal intake of carbohydrates (<1.2 g/kg/day) can heighten muscle glycogen recovery, and may help mitigate changes in muscle damage markers.
- Total protein and calorie intake appears to be the most important consideration when it comes to promoting positive adaptations to resistance training, and the impact of timing strategies (immediately before or immediately after) to heighten these adaptations in non-athletic populations appears to be minimal.

## Recommended intake

Proteins provide the building blocks of all tissues via their constituent amino acids. Athletes consume dietary protein to repair and rebuild skeletal muscle and connective tissues following intense training bouts or athletic events. During in the 1980s and early 1990’s Tarnopolsky [129], Phillips [130], and Lemon [131] first demonstrated that total protein needs were 50 to 175% greater in athletes than sedentary controls. A report in 2004 by Phillips [132] summarized the findings surrounding protein requirements in resistance-trained athletes. Using a regression approach, he concluded that a protein intake of 1.2 g of protein per kg of body weight per day (g/kg/day) should be recommended, and when the upper limit of a 95% confidence interval was included the amount approached 1.33 g/kg/day. A key consideration regarding these recommended values is that all generated data were obtained using the nitrogen balance technique, which is known to underestimate protein requirements. Interestingly, two of the included papers had prescribed protein intakes of 2.4 and 2.5 g/kg/day, respectively [129, 133]. All data points from these two studies also had the highest levels of positive nitrogen balance. For an athlete seeking to ensure an anabolic environment, higher daily protein intakes might be needed. Another challenge that underpins the ability to universally and successfully recommend daily protein amounts are factors related to the volume of the exercise program, age, body composition and training status of the athlete; as well as the total energy intake in the diet, particularly for athletes who desire to lose fat and are restricting calories to accomplish this goal [134]. For these reasons, and due to an increase of published studies in areas related to optimal protein dosing, timing and composition, protein needs are being recommended within this position stand on a per meal basis.

For example, Moore [31] found that muscle and albumin protein synthesis was optimized at approximately 20 g of egg protein at rest. Witard et al. [135] provided incremental doses of whey protein (0, 10, 20 and 40 g) in conjunction with an acute bout of resistance exercise and concluded that a minimum protein dose of 20 g optimally promoted MPS rates. Finally, Yang and colleagues [136] had 37 elderly men (average age of 71 years) consume incremental doses of whey protein isolate (0, 10, 20 and 40 g/dose) in combination with a single bout of lower body resistance exercise and concluded that a 40 g dose of whey protein isolate is needed in this population to maximize rates of MPS. Furthermore, while results from these studies offer indications of what optimal absolute dosing amounts may be, Phillips [134] concluded that a relative dose of 0.25 g of protein per kg of body weight per dose might operate as an optimal supply of high-quality protein. Once a total daily target protein intake has been achieved, the frequency and pattern with which optimal doses are ingested may serve as a key determinant of overall changes in protein synthetic rates.

Research indicates that rates of MPS rapidly rise to peak levels within 30 min of protein ingestion and are maintained for up to three hours before rapidly beginning to lower to basal rates of MPS even though amino acids are still elevated in the blood [137]. Using an oral ingestion model of 48 g of whey protein in healthy young men, rates of myofibrillar protein synthesis increased three-fold within 45–90 min before slowly declining to basal rates of MPS all while plasma concentration of EAAs remained significantly elevated [138]. While human models have not fully explored the mechanistic basis of this ‘muscle-full’ phenomenon, an energy deficit theory has been proposed which hypothesizes that rates of MPS were blunted even though plasma concentrations of amino acids remained elevated because a relative lack of cellular ATP was available to drive the synthetic process [139]. While largely unexplored in a human model, these authors relied upon an animal model and were able to reinstate increases in MPS using the consumption of leucine and carbohydrate 135 min after ingestion of the first meal. As such, it is suggested that individuals attempting to restrict caloric intake should consume three to four whole meals consisting of 20–40 g of protein per meal. While this recommendation stems primarily from initial work that indicated protein doses of 20–40 g favorably promote increased rates of MPS [31, 135, 136], Kim and colleagues [140] recently reported that a 70 g dose of protein promoted a more favorable net balance of protein when compared to a 40 g dose due to a stronger attenuation of rates of muscle protein breakdown.

For those attempting to increase their calories, we suggest consuming small snacks between meals consisting of both a complete protein and a carbohydrate source. This contention is supported by research from Paddon-Jones et al. [97] that used a 28-day bed rest model. These researchers compared three 850-cal mixed macronutrient meals to three 850-cal meals combined with three 180-cal amino acid-carbohydrate snacks between meals. Results demonstrated that subjects, who also consumed the small snacks, experienced a 23% increase in muscle protein fractional synthesis and successful maintenance of strength throughout the bed rest trial. Additionally, using a protein distribution pattern of 20–25 g doses every three hours in response to a single bout of lower body resistance exercise appears to promote the greatest increase in MPS rates and phosphorylation of key intramuscular proteins linked to muscle hypertrophy [126]. Finally, in a series of experiments, Arciero and colleagues [116, 141] employed a protein pacing strategy involving equitable distribution of effective doses of protein (4–6 meals/day of 20–40 g per meal) alone and combined with multicomponent exercise training. Using this approach, their results consistently demonstrate positive changes in body composition [116, 142] and physical performance outcomes in both lean [143, 144] and overweight/obese populations [142, 143, 145]. This simple addition could provide benefits for individuals looking to increase muscle mass and improve body composition in general while also striving to maintain or improve health and performance.

### Key points

- The current RDA for protein is 0.8 g/kg/day with multiple lines of evidence indicating this value is not an appropriate amount for a training athlete to meet their daily needs.
- While previous recommendations have suggested a daily intake of 1.2–1.3 g/kg/day is an appropriate amount, most of this work was completed using the nitrogen balance technique, which is known to systematically underestimate protein needs.
- Daily and per dose needs are combinations of many factors including volume of exercise, age, body composition, total energy intake and training status of the athlete.
- Daily intakes of 1.4 to 2.0 g/kg/day operate as a minimum recommended amount while greater amounts may be needed for people attempting to restrict energy intake while maintaining fat-free mass.
- Recommendations regarding the optimal protein intake per serving for athletes to maximize MPS are mixed and are dependent upon age and recent resistance exercise stimuli. General recommendations are 0.25 g of a high-quality protein per kg of body weight, or an absolute dose of 20–40 g.
- Higher doses (~40 g) are likely needed to maximize MPS responses in elderly individuals.
- Even higher amounts (~70 g) appear to be necessary to promote attenuation of muscle protein breakdown.
- Pacing or spreading these feeding episodes approximately three hours apart has been consistently reported to promote sustained, increased levels of MPS and performance benefits.

## Protein quality

There are 20 total amino acids, comprised of 9 EAAs and 11 non-essential amino acids (NEAAs). EAAs cannot be produced in the body and therefore must be consumed in the diet. Several methods exist to determine protein quality such as Chemical Score, Protein Efficiency Ratio, Biological Value, Protein Digestibility-Corrected Amino Acid Score (PDCAAS) and most recently, the Indicator Amino Acid Oxidation (IAAO) technique. Ultimately, in vivo protein quality is typically defined as how effective a protein is at stimulating MPS and promoting muscle hypertrophy [146]. Overall, research has shown that products containing animal and dairy-based proteins contain the highest percentage of EAAs and result in greater hypertrophy and protein synthesis following resistance training when compared to a vegetarian protein-matched control, which typically lacks one or more EAAs [86, 93, 147].

Several studies, but not all, [148] have indicated that EAAs alone stimulate protein synthesis in the same magnitude as a whole protein with the same EAA content [98]. For example, Borsheim et al. [52] found that 6 g of EAAs stimulated protein synthesis twice as much as a mixture of 3 g of NEAAs combined with 3 g of EAAs. Moreover, Paddon-Jones and colleagues [96] found that a 180-cal supplement containing 15 g of EAAs stimulated greater rates of protein synthesis than an 850-cal meal with the same EAA content from a whole protein source. While important, the impact of a larger meal on changes in circulation and the subsequent delivery of the relevant amino acids to the muscle might operate as important considerations when interpreting this data. In contrast, Katsanos and colleagues [148] had 15 elderly subjects consume either 15 g of whey protein or individual doses of the essential and nonessential amino acids that were identical to what is found in a 15-g whey protein dose on separate occasions. Whey protein ingestion significantly increased leg phenylalanine balance, an index of muscle protein accrual, while EAA and NEAA ingestion exerted no significant impact on leg phenylalanine balance. This study, and the results reported by others [149] have led to the suggestion that an approximate 10 g dose of EAAs might serve as an optimal dose to maximally stimulate MPS and that intact protein feedings of appropriate amounts (as opposed to free amino acids) to elderly individuals may stimulate greater improvements in leg muscle protein accrual.

Based on this research, scientists have also attempted to determine which of the EAAs are primarily responsible for modulating protein balance. The three branched-chain amino acids (BCAAs), leucine, isoleucine, and valine are unique among the EAAs for their roles in protein metabolism [150], neural function [151–153], and blood glucose and insulin regulation [154]. Additionally, enzymes responsible for the degradation of BCAAs operate in a rate-limiting fashion and are found in low levels in splanchnic tissues [155]. Thus, orally ingested BCAAs appear rapidly in the bloodstream and expose muscle to high concentrations ultimately making them key components of skeletal MPS [156]. Furthermore, Wilson and colleagues [157] have recently demonstrated, in an animal model, that leucine ingestion (alone and with carbohydrate) consumed between meals (135 min post-consumption) extends protein synthesis by increasing the energy status of the muscle fiber. Multiple human studies have supported the contention that leucine drives protein synthesis [158, 159]. Moreover, this response may occur in a dose-dependent fashion, plateauing at approximately two g at rest [31, 157], and increasing up to 3.5 g when ingestion occurs after completion of a 60-min bout of moderate intensity cycling [159]. However, it is important to realize that the duration of protein synthesis after resistance exercise appears to be limited by both the signal (leucine concentrations), ATP status, as well as the availability of substrate (i.e., additional EAAs found in a whole protein source) [160]. As such, increasing leucine concentration may stimulate increases in muscle protein, but a higher total dose of all EAAs (as free form amino acids or intact protein sources) seems to be most suited for sustaining the increased rates of MPS [160].

It is well known that exercise improves net muscle protein balance and in the absence of protein feeding, this balance becomes more negative. When combined with protein feeding, net muscle protein balance after exercise becomes positive [161]. Norton and Layman [150] proposed that consumption of leucine, could turn a negative protein balance to a positive balance following an intense exercise bout by prolonging the MPS response to feeding. In support, the ingestion of a protein or essential amino acid complex that contains sufficient amounts of leucine has been shown to shift protein balance to a net positive state after intense exercise training [46, 150]. Even though leucine has been demonstrated to independently stimulate protein synthesis, it is important to recognize that supplementation should not be with just leucine alone. For instance, Wilson et al. [139] demonstrated in an animal model that leucine consumption resulted in a lower duration of protein synthesis compared to a whole meal. In summary, athletes should focus on consuming adequate leucine content in each of their meals through selection of high-quality protein sources [139].

### Key points

- Protein sources containing higher levels of the EAAs are considered to be higher quality sources of protein.
- The body uses 20 amino acids to make proteins, seven of which are essential (nine conditionally), requiring their ingestion to meet daily needs.
- EAAs appear to be uniquely responsible for increasing MPS with doses ranging from 6 to 15 g all exerting stimulatory effects. In addition, doses of approximately one to three g of leucine per meal appear to be needed to stimulate protein translation machinery.
- The BCAAs (i.e., isoleucine, leucine, and valine) appear to exhibit individual and collective abilities to stimulate protein translation. However, the extent to which these changes are aligned with changes in MPS remains to be fully explored.
- While greater doses of leucine have been shown to independently stimulate increases in protein synthesis, a balanced consumption of the EAAs promotes the greatest increases.
- The prioritization of feedings of protein with adequate levels of leucine/BCAAs will best promote increases in MPS.

## Protein sources

### Milk proteins

Milk proteins have undergone extensive research related to their potential roles in augmenting adaptations from exercise training [86, 93]. For example, consuming milk following exercise has been demonstrated to accelerate recovery from muscle damaging exercise [162], increase glycogen replenishment [163], improve hydration status [162, 164], and improve protein balance to favor synthesis [86, 93], ultimately resulting in increased gains in both neuromuscular strength and skeletal muscle hypertrophy [93]. Moreover, milk protein contains the highest score on the PDCAAS rating system, and in general contains the greatest density of leucine [156]. Milk can be fractionated into two protein classes, casein and whey.

Comparison of the quality of whey and casein reveal that these two proteins routinely contain the highest leucine content of all other protein sources at 11% and 9.3%, respectively. While both are high in quality, the two differ in the rate at which they digest as well as the impact they have on protein metabolism [165–167]. Whey protein is water soluble, mixes easily, and is rapidly digested [168]. In contrast, casein is water insoluble, coagulates in the gut and is digested more slowly than whey protein [168]. Casein also has intrinsic properties such as opioid peptides, which effectively slow gastric motility [168]. Original research investigating the effects of digestion rate was conducted by Boirie, Dangin and colleagues [165–167]. These researchers gave a 30 g bolus of whey protein and a 43 g bolus of casein protein to subjects on separate occasions and measured amino acid levels for several hours after ingestion. They reported that the whey protein condition displayed robust hyperaminoacidemia 100 min after administration. However, by 300 min, amino acid concentrations had returned to baseline. In contrast, the casein condition resulted in a slow increase in amino acid concentrations, which remained elevated above baseline after 300 min. Over the study duration, casein produced a greater whole body leucine balance than the whey protein condition, leading the researcher to suggest that prolonged, moderate hyperaminoacidemia is more effective at stimulating increases in whole body protein anabolism than a robust, short lasting hyperaminoacidemia.

While this research appears to support the efficacy of slower digesting proteins, subsequent work has questioned its validity in athletes. The first major criticism is that Boire and colleagues investigated whole body (non-muscle and muscle) protein balance instead of skeletal (myofibrillar) MPS. This is important considering that skeletal muscle protein turnover occurs at a much slower rate than protein turnover of both plasma and gut proteins; as a result, MPS has been suggested to contribute anywhere from 25 to 50% of total whole body protein synthesis [169]. These findings suggest that changes in whole body protein turnover may poorly reflect the level of skeletal muscle protein metabolism that may be taking place. Trommelen and investigators [121] examined 24 young men ingesting 30 g of casein protein with or without completion of a single bout of resistance exercise, and concluded that rates of MPS were increased, but whole-body protein synthesis rates were not impacted.

More recently, Tang and colleagues [86] investigated the effects of administering 22 g of hydrolyzed whey isolate and micellar casein (10 g of EAAs) at both rest and following a single bout of resistance training in young males. The area under the curve calculations demonstrated a 200% greater increase in leucine concentrations in the blood following whey versus casein ingestion. Moreover, these researchers reported that whey protein ingestion stimulated greater MPS at both rest and following exercise when compared to casein. Tipton et al. [79] used an acute study design involving a single bout of lower body resistance exercise and 20-g doses of casein or whey after completing the exercise session. In comparison to the control group, both whey and casein significantly increased leucine balance, but no differences were found between the two protein sources for amino acid uptake and muscle protein balance. Additional research has also demonstrated that 10 weeks of whey protein supplementation in trained bodybuilders resulted in greater gains in lean mass (5.0 vs. 0.8 kg) and strength compared to casein [170]. These findings suggest that the faster-digesting whey proteins may be more beneficial for skeletal muscle adaptations than the slower digesting casein.

### Effects of milk proteins on glycogen replenishment and skeletal muscle damage

Skeletal muscle glycogen stores are a critical element to both prolonged and high-intensity exercise. In skeletal muscle, glycogen synthase activity is considered one of the key regulatory factors for glycogen synthesis. Research has demonstrated that the addition of protein in the form of milk and whey protein isolate (0.4 g/kg) to a moderate (0.8 g/kg), but not high (1.2 g/kg) carbohydrate-containing (dextrose-maltodextrin) beverage promotes increased rates of muscle glycogen replenishment following hard training [47]. Further, the addition of protein facilitates repair and recovery of the exercised muscle [12]. These effects are thought to be related to a greater insulin response following the exercise bout. Intriguingly, it has also been demonstrated that whey protein enhances glycogen synthesis in the liver and skeletal muscle more than casein in an insulin-independent fashion that appears to be due to its capacity to upregulate glycogen synthase activity [171]. Therefore, the addition of milk protein to a post-workout meal may augment recovery, improve protein balance, and speed glycogen replenishment.

### Health benefits of milk-based proteins

While athletes tend to view whey as the ideal protein for skeletal muscle repair and function it also has several health benefits. In particular, whey protein contains an array of biologically active peptides whose amino acids sequences give them specific signaling effects when liberated in the gut. Not only is whey protein high in β-Lactoglobulin and α-lactalbumin (75% of total bovine whey proteins), but it is also rich in EAAs (approximately 50% by weight). Furthermore, whey protein appears to play a role in enhancing lymphatic and immune system responses [106]. In addition, α-lactalbumin contains an ample supply of tryptophan which increases cognitive performance under stress [172], improves the quality of sleep [172, 173], and may also speed wound healing [172], properties which could be vital for recovery from combat and contact sporting events. In addition, lactoferrin is also found in both milk and in whey protein, and has been demonstrated to have antibacterial, antiviral, and antioxidant properties [174]. Moreover, there is some evidence that whey protein can bind iron and therefore increase its absorption and retention [175].

### Egg proteins

Egg protein is often thought of as an ideal protein because its amino acid profile has been used as the standard for comparing other dietary proteins [168]. Due to their excellent digestibility and amino acid content, eggs are an excellent source of protein for athletes. While the consumption of eggs has been criticized due to their cholesterol content, a growing body of evidence demonstrates the lack of a relationship between egg consumption and coronary heart disease, making egg-based products more appealing [176]. One large egg has 75 kcal and 6 g of protein, but only 1.5 g of saturated fat while one large egg white has 16 kcal with 3.5 g of protein and is fat-free. Research using eggs as the protein source for athletic performance and body composition is lacking, perhaps due to less funding opportunities relative to funding for dairy. Egg protein may be particularly important for athletes, as this protein source has been demonstrated to significantly increase protein synthesis of both skeletal muscle and plasma proteins after resistance exercise at both 20 and 40 g doses. Leucine oxidation rates were found to increase following the 40 g dose, suggesting that this amount exceeds an optimal dose [31]. In addition to providing a cost effective, high-quality source of protein rich in leucine (0.5 g of leucine per serving), eggs have also been identified as a functional food [177]. Functional foods are defined as foods that, by the presence of physiologically active components, provide a health benefit beyond basic nutrition [178]. According to the Academy of Nutrition and Dietetics, functional foods should be consumed as part of a varied diet on a regular basis, at effective levels [179]. Thus, it is essential that athletes select foods that meet protein requirements and also optimize health and prevent decrements in immune function following intense training. Important nutrients provided by eggs include riboflavin (15% RDA), selenium (17% RDA) and vitamin K (31% RDA) [177]. Eggs are also rich in choline, a nutrient which may have positive effects on cognitive function [180]. Moreover, eggs provide an excellent source of the carotenoid-based antioxidants lutein and zeaxanthin [181]. Also, eggs can be prepared with most meal choices, whether at breakfast, lunch, or dinner. Such positive properties increase the probability of the athletes adhering to a diet rich in egg protein.

### Beef and other flesh proteins

Meat proteins are a major staple in the American diet and, depending on the cut of meat, contain varying amounts of fat and cholesterol. Meat proteins are well known to be rich sources of the EAAs [182]. Beef is a common source of dietary protein and is considered to be of high biological value because it contains the full balance of EAAs in a fraction similar to that found in human skeletal muscle [182]. A standard serving of 113.4 g lean beef provides 10 g of the EAAs (3.5 g of leucine) and 30 g of total amino acids. Moreover, this 30 g dose of beef protein has been shown to stimulate protein synthesis in both young and elderly subjects [182]. In addition to its rich content of amino acids, beef and other flesh proteins can serve as important sources of micronutrients such as iron, selenium, vitamins A, B12 and folic acid. For the most part, these quality minerals and micronutrients cannot be as easily obtained through plant-based proteins and/or the bioavailability of these macronutrients from plants is limited. This is a particularly important consideration for pregnant and breastfeeding women. Ultimately, as an essential part of a mixed diet, meat helps to ensure adequate distribution of essential micronutrients and amino acids to the body.

Research has shown that significant differences in skeletal muscle mass and body composition between older men who resistance train and either consume meat-based or lactoovovegetarian diet [147]. Over a 12-week period, whole-body density, fat-free mass, and whole-body muscle mass (as measured by urinary creatinine excretion) increased in the meat-sourced diet group but decreased in the lactoovovegetarian diet group. These results indicate that not only do meat-based diets increase fat-free mass, but also they may specifically increase muscle mass, thus supporting the many benefits of meat-based diets. A diet high in meat protein in older adults may provide an important resource in reducing the risk of sarcopenia.

Positive results have also been seen in elite athletes that consume meat-based proteins, as opposed to vegetarian diets [183]. For example, carnitine is a molecule that transports long-chain fatty acids into mitochondria for oxidation and is found in high amounts in meat. While evidence is lacking to support an increase in fat oxidation with increased carnitine availability, carnitine has been linked to the sparing of muscle glycogen, and decreases in exercise-induced muscle damage [184]. Certainly, more research is needed to support these assertions. Creatine is a naturally occurring compound found mainly in muscle. The concentration of creatine in uncooked chicken and beef is approximately 30 mmol/kg (4–5 g/kg), meaning that one serving of beef contains approximately 0.4 g of creatine [185]. Vegetarians have lower total body creatine stores than omnivores, which demonstrates that regular meat eating has a significant effect on human creatine status [186]. Moreover, creatine supplementation studies with vegetarians indicate that increased creatine uptake levels do exist in people who practice various forms of vegetarianism [187]. Sharp and investigators [188] published the only study known to compare different supplemental (powdered) forms of animal proteins on adaptations to resistance training such as increases in strength and improvements in body composition. Forty-one men and women performed a standardized resistance-training program over eight weeks and consumed a daily 46 g dose of either hydrolyzed chicken protein, beef protein isolate, or whey protein concentrate in comparison to a control group. All groups experienced similar increases in upper and lower-body strength, but all protein-supplemented groups reported significant increases in lean mass and decreases in fat mass.

Meat-based diets have been shown to include additional overall health benefits. Some studies have found that meat, as a protein source, is associated with higher serum levels of IGF-1 [189], which in turn is related to increased bone mineralization and fewer fractures [190].

### Meat vs. plant based proteins: Is one better than the other?

A highly debated topic in nutrition and epidemiology is whether vegetarian diets are a healthier choice than omnivorous diets. One key difference is the fact that vegetarian diets often lack equivalent amounts of protein when compared to omnivorous diets [147]. However, with proper supplementation and careful nutritional choices, it is possible to have complete proteins in a vegetarian diet. Generally by consuming high-quality, animal-based products (meat, milk, eggs, and cheese) an individual will achieve optimal growth as compared to ingesting only plant proteins [147]. Research has shown that soy is considered a lower quality complete protein. Hartman et al. [93] had participants consume a mixture of sucrose and either 30 g of milk or soy proteins during 12-weeks of resistance training. They found that the participants that consumed the milk protein increased lean mass and decreased fat mass more than the control and soy groups. Moreover, the soy group was not significantly different from the control group. Similarly, a study by Tang and colleagues [86] directly compared the abilities of hydrolyzed whey isolate, soy isolate, and micellar casein to stimulate rates of MPS both at rest and in response to a single bout of lower body resistance training. These authors reported that the ability of soy to stimulate MPS was greater than casein, but less than whey, at rest and in response to an acute resistance exercise stimulus. While soy is considered a complete protein, it contains lower amounts of BCAAs than bovine milk [168]. Additionally, research has found that dietary soy phytoestrogens inhibit mTOR expression in skeletal muscle through activation of AMPK [191]. Thus, not only does soy contain lower amounts of the EAAs and leucine, but soy protein may also be responsible for inhibiting growth factors and protein synthesis via its negative regulation of mTOR. When considering the multitude of plant sources of protein, soy overwhelmingly has the most research. Limited evidence using wheat protein in older men has suggested that wheat protein stimulates significantly lower levels of MPS when compared to an identical dose (35 g) of casein protein, but when this dose is increased nearly two fold (60 g) this protein source is able to significantly increase rates of myofibrillar protein synthesis [192]. Rice protein is a medium to slow absorbing protein, which is in line with other non-meat/non-dairy proteins, however, leucine from rice protein shows unique absorption kinetics, peaking faster than leucine from whey protein [193]. As mentioned earlier, a study by Joy and colleagues [89] in which participants participated in resistance training program for eight weeks while taking identical, high doses of either rice or whey protein, demonstrated that rice protein stimulated similar increases in body composition adaptations to whey protein.

### Protein blends

The majority of available science has explored the efficacy of ingesting single protein sources, but evidence continues to mount that combining protein sources may afford additional benefits [194]. For example, a 10-week resistance training study by Kerksick and colleagues [22] demonstrated that a combination of whey (40 g) and casein (8 g) yielded the greatest increase in fat-free mass (determined by DEXA) when compared to both a combination of 40 g of whey, 5 g of glutamine, and 3 g of BCAAs and a placebo consisting of 48 g of a maltodextrin carbohydrate. Later, Kerksick et al. [95] demonstrated various combinations of whey, casein, and colostrum proteins with and without creatine can also yield positive improvements in strength and body composition over a 12-week resistance training and supplementation regimen. Similarly, Hartman and investigators [93] had 56 healthy young men train for 12 weeks while either ingesting isocaloric and isonitrogenous doses of fat-free milk (a blend of whey and casein), soy protein or a carbohydrate placebo and concluded that fat-free milk stimulated the greatest increases in Type I and II muscle fiber area as well as fat-free mass; however, strength outcomes were not affected. Moreover, Wilkinson and colleagues [94] demonstrated that ingestion of fat-free milk (vs. soy or carbohydrate) led to a greater area under the curve for net balance of protein and that the fractional synthesis rate of muscle protein was greatest after milk ingestion. In 2013, Reidy et al. [195] indicated that a mixture of whey and soy protein over a four-hour measurement window similarly increased MPS rates during the early (0–2 h) time-period versus whey protein, but only the protein blend was able to stimulate significantly increased MPS rates during the later (2–4 h) measurement window. However, when the entire four-hour measurement period was considered, no difference in MPS rates were found. A follow-up publication from the same clinical trial also reported that ingestion of the protein blend resulted in a positive and prolonged amino acid balance when compared to ingestion of whey protein alone, while post-exercise rates of myofibrillar protein synthesis were similar between the two conditions [196]. Reidy et al. [197] reported that in 68 healthy young men who were participating in a supervised resistance-training program over 12 weeks, there were increases in whole body lean mass with either whey protein or a whey protein and soy protein blend compared to a maltodextrin placebo. No differences were found between whey and the whey and soy blend.

### Criteria for comparing protein sources

Some valid criteria exist to compare protein sources and provide an objective method of how to include them in a diet. As previously mentioned, common means of assessing protein quality include Biological Value, Protein Efficiency Ratio, PDCAAS and IAAO. The derivation of each technique is different with all having distinct advantages and disadvantages. For nearly all populations, ideal methods should be linked to the capacity of the protein to positively affect protein balance in the short term, and facilitate increases and decreases in lean and fat-mass, respectively, over the long term. In addition, the protein’s ability to enhance immune function and promote an anti-oxidative environment should also be considered. To this point, dairy, egg, meat, and plant-based proteins have been discussed. Two critical variables exist that determine a protein’s impact on overall protein accretion and protein turnover: a) the protein’s leucine content and b) the rate at which the protein is digested. In general, the proteins with the greatest leucine content include dairy (9–11%), egg (8.6%), and meat (8%), while sources low in leucine include plant-based proteins. Faster digesting sources of protein include whey and egg whites, soy, and very lean cuts of meat (>95% lean). In contrast, casein and fatty cuts of meat (<80% lean) act as slowly digested sources of protein. As mentioned previously, initial research by Boirie and Dangin has highlighted the impact of protein digestion rate on net protein balance with the two milk proteins: whey and casein [165–167]. Subsequent follow-up work has used this premise as a reference point for the digestion rates of other protein sources.

Using the criteria of leucine content, Norton and Wilson et al. [198, 199] used animal models to compare the potential to activate initiation factors and MPS between four different protein sources: wheat (supplemented with leucine), soy, egg, and whey, (containing 6.8, 8.0, 8.8, and 11% leucine, respectively) using a diet consisting of three meals per day. Macronutrient intake was 16/54/30% for protein, carbohydrates and fat, respectively. Wheat and soy did not stimulate MPS above fasted levels, whereas egg and whey proteins significantly increased MPS rates, with MPS for whey protein being greater than egg protein. MPS responses were closely related to changes in plasma leucine and phosphorylation of 4E–BP1 and S6 K protein signaling molecules. More importantly, following 2- and 11-weeks of ingestion, it was demonstrated that the leucine content of the meals increased muscle mass and was inversely correlated with body fat.

Tang et al. [86] compared high leucine/fast-digesting (hydrolyzed whey isolate), lower leucine/intermediate digesting (soy isolate) and high leucine/slow-digesting (micellar casein) protein sources on MPS at rest and following exercise. The researchers demonstrated that MPS at rest was higher after ingestion of faster digesting proteins compared to slower digesting proteins (whey and soy > casein). Specifically, MPS after consumption of whey was approximately 93% and 18% greater than casein and soy, respectively. A similar pattern of results was observed after resistance exercise (whey > soy > casein) whereby protein synthesis following whey consumption was approximately 122% and 31% greater than casein and soy, respectively. MPS was also greater after soy consumption at rest (64%) and following resistance exercise (69%) compared with casein. These findings lead us to conclude that athletes should seek protein sources that are both fast-digesting and high in leucine content to maximally stimulate rates of MPS at rest and following training. Moreover, in consideration of the various additional attributes that high-quality protein sources deliver, it may be advantageous to consume a combination of higher quality protein sources (dairy, egg, and meat sources).

#### Key points

- Multiple protein sources are available for an athlete to consider, and each has their own advantages and disadvantages.
- Protein sources are commonly evaluated based upon the content of amino acids, particularly the EAAs, they provide. Beyond amino acid content, the fat, calorie, and micronutrient content, and presence of various bioactive peptides all contribute to a protein’s quality.
- Leucine content and rate of digestion have also been demonstrated in multiple scientific studies to play an important role in an athlete’s ability to train, compete, and recover.
- Blends of protein sources might afford a favorable combination of key nutrients such as leucine, EAAs, bioactive peptides, and antioxidants, but more research is needed to determine their ideal composition.

## Preparation methods of various proteins

Nutrient density is defined as the amount of a particular nutrient (carbohydrate, protein, fat, etc.) per unit of energy in a given food. In many situations, the commercial preparation method of foods can affect the actual nutrient density of the resulting food. Using protein as an example, full-fat milk is approximately 150 cal a serving, and of this 8 g, or about 21% is from protein. Skim milk on the other hand contains approximately 9 g of protein in a 90-cal eight-ounce serving, making it approximately 40% protein. When producing milk protein supplements, special preparations must be made to separate the protein sources from the lactose and fat calories in milk. For example, the addition of acid to milk causes the casein to coagulate or collect at the bottom, while the whey is left on the top [200]. These proteins are then filtered to increase their purity. A concentrate is commonly defined as any protein product that is 29–80% protein by dry weight. Sport nutrition products generally use concentrates that are 70–80% protein [200]. As extra filtering steps are added, the purity of the final product increases and when a final protein product yields greater than 90% protein, it is considered an isolated protein [200].

### Filtration processes

Filtration methods differ, and there are both benefits and disadvantages to each. The two most popular methods of filtration of a given protein are the use of ion exchange and micro/ultrafiltration methods. Ion exchange exposes a given protein source, such as whey, to hydrochloric acid and sodium hydroxide, thereby producing an electric charge on the proteins that can be used to separate them from lactose and fat [200]. The advantage of this method is that it is relatively cheap and produces the highest protein concentration [200]. The disadvantage is that ion exchange filtration typically denatures some of the valuable immune-boosting, anti-carcinogenic peptides found in whey [200]. Cross-flow microfiltration, and ultra-micro filtration are based on the premise that the molecular weight of whey protein is greater than lactose, and use 1 and 0.25-μm ceramic membranes, respectively, to separate the two. As a result, whey protein is trapped in the membranes but the lactose and other components pass through. The advantage is that these processes do not denature valuable proteins and peptides found in whey, so the protein itself is deemed to be of higher quality [200]. The main disadvantage is that this filtration process is typically costlier than the ion exchange method.

### Hydrolyzed proteins

When consumed whole, proteins are digested through a series of steps beginning with homogenization by chewing, followed by partial digestion by pepsin in the stomach [201]. Following this, a combination of peptides, proteins, and negligible amounts of single amino acids are released into the small intestine and from there are either partially hydrolyzed into oligopeptides, 2–8 amino acids in length or are fully hydrolyzed into individual amino acids [201]. Absorption of individual amino acids and various small peptides (di, tri, and tetra) into the blood occurs inside the small intestine through separate transport mechanisms [201]. Oftentimes, products contain proteins that have been pre-exposed to specific digestive enzymes causing hydrolysis of the proteins into di, tri, and tetrapeptides. A plethora of studies have investigated the effects of the degree of protein fractionation (or degree of hydrolysis) on the absorption of amino acids and the subsequent hormonal response [202–207]. Research indicates that amino acids are absorbed more rapidly when they are consumed as di and/or tri peptides compared to free form amino acids or complete proteins [205]. Further, the rate of absorption may lead to a more favorable anabolic hormonal environment [202, 203, 206]. Calbet et al. [203] examined both amino acid appearance and insulin responses following consumption of protein solutions containing the same amount of protein, or pure carbohydrates. The treatments consisted of a pure glucose solution, whey peptide hydrolysates, and cow’s milk containing milk proteins, lactose and fat. Each of the nitrogen containing solutions contained 15 g of glucose and 30 g of protein. Results indicated that peptide hydrolysates produced a faster increase in venous plasma amino acids compared to milk proteins. Further, the peptide hydrolysates produced peak plasma insulin levels that were two- and four-times greater than that evoked by the milk and glucose solutions, respectively, with a correlation of 0.8 between plasma amino acids and the insulin response in the peptide hydrolysates. One of the inherent shortcomings of this study is that milk proteins are 80% casein and, therefore, are not ideal candidates to compare with hydrolyzed whey.

In a more appropriate comparison, Morifuji et al. [205] investigated the effects of 12.5 g of either hydrolyzed or non-hydrolyzed soy and whey proteins on changes in plasma levels of the EAAs, BCAAs, and insulin. Results indicated that protein hydrolysates produced greater responses than their non-hydrolyzed counterpart in plasma for each of the variables (Hydrolyzed whey > Non-hydrolyzed whey > hydrolyzed soy > Non-hydrolyzed soy). However, Calbet et al. [202] found that 36 g of hydrolyzed or non-hydrolyzed whey and casein led to no differences in the plasma amino acid/BCAA responses in the whey groups. The hydrolyzed casein, however, did result in a greater amino acid response than the nonhydrolyzed casein. Finally, both hydrolyzed groups resulted in greater gastric secretions, as well as greater plasma increases, in glucose-dependent insulinotropic polypeptides [208].

Buckley and colleagues [207] found that a ~ 30 g dose of a hydrolyzed whey protein isolate resulted in a more rapid recovery of muscle force-generating capacity following eccentric exercise, compared with a flavored water placebo or a non-hydrolyzed form of the same whey protein isolate. Indeed, the effect of this hydrolysate was such that complete recovery of muscle force-generating capacity had been achieved by six hours post supplementation, while the normal whey and placebo groups’ strength remained depressed 24 h later. In agreement with these findings, Cooke et al. [209] had 17 untrained men complete an eccentric-based resistance training bout to invoke muscle damage and supplemented with either carbohydrate or a hydrolyzed whey protein isolate. Three and seven days after completing the damaging exercise bout, maximal strength levels were higher in the hydrolyzed whey protein group compared to carbohydrate supplementation. Additionally, blood concentrations of muscle damage markers tended to be lower when four ~30-g doses of a hydrolyzed whey protein isolate were ingested for two weeks following the damaging bout. Beyond influencing strength recovery after damaging exercise, other benefits of hydrolyzed proteins have been suggested. For example, Morifuji et al. [210] using an animal model reported that the ability of whey hydrolysates to increase skeletal muscle glycogen replenishment after exercise was greater when compared to BCAA ingestion. Furthermore, Lockwood et al. [204] investigated the effects of ingesting either 30 g of hydrolyzed whey or two varying forms of whey protein concentrates during a linear resistance-training protocol over 8 weeks. Results indicated that strength and lean body mass (LBM) increased equally in all groups. However, fat mass decreased only in the hydrolyzed whey protein group. While more work needs to be completed to fully determine the potential impact of hydrolyzed proteins on strength and body composition changes, this initial study suggests that hydrolyzed whey may be efficacious for decreasing body fat. Finally, Saunders et al. [7] had thirteen trained male cyclists complete a simulated 60-km time trial where they ingested either carbohydrate or carbohydrate and protein hydrolysate at equal intervals throughout the race as well as at the conclusion of the race. The authors reported that co-ingestion of a carbohydrate and protein hydrolysate improved time-trial performance late in the exercise protocol and significantly reduced soreness and markers of muscle damage. Two excellent reviews on the topic of hydrolyzed proteins and their impact on performance and recovery have been published by Van Loon et al. [211] and Saunders [212].

### Digestive enzymes in proteins

Digestion is the physiological process of rendering the food we eat into smaller components that allow key nutrients to be assimilated into our body’s tissues. The prevalence of digestive enzymes in sports nutrition products has increased during recent years with many products now containing a combination of proteases and lipases, with the addition of carbohydrates in plant proteins. Proteases can hydrolyze proteins into various peptide configurations and potentially single amino acids. It appears that digestive enzyme capabilities and production decrease with age [213], thus increasing the difficulty with which the body can break down and digest large meals. Digestive enzymes could potentially work to promote optimal digestion by allowing up-regulation of various metabolic enzymes that may be needed to allow for efficient bodily operation. Further, digestive enzymes have been shown to minimize quality differences between varying protein sources [214]. Individuals looking to increase plasma peak amino acid concentrations may benefit from hydrolyzed protein sources or protein supplemented with digestive enzymes. However, more work is needed before definitive conclusions can be drawn regarding the efficacy of digestive enzymes.

## Protein safety

Despite a plethora of studies demonstrating safety, much concern still exists surrounding the clinical implications of consuming increased amounts of protein, particularly on renal and hepatic health. The majority of these concerns stem from renal failure patients and educational dogma that has not been rewritten as evidence mounts to the contrary. Certainly, it is clear that people in renal failure benefit from protein-restricted diets [215], but extending this pathophysiology to otherwise healthy exercise-trained individuals who are not clinically compromised is inappropriate. Published reviews on this topic consistently report that an increased intake of protein by competitive athletes and active individuals provides no indication of hepato-renal harm or damage [216, 217]. This is supported by a recent commentary [134] which referenced recent reports from the World Health Organization [218] where they indicated a lack of evidence linking a high protein diet to renal disease. Likewise, the panel charged with establishing reference nutrient values for Australia and New Zealand also stated there was no published evidence that elevated intakes of protein exerted any negative impact on kidney function in athletes or in general [219].

Recently, Antonio and colleagues published a series of original investigations that prescribed extremely high amounts of protein (~3.4–4.4 g/kg/day) and have consistently reported no harmful effects [220–223]. The first study in 2014 had resistance-trained individuals consume an extremely high protein diet (4.4 g/kg/day) for eight weeks and reported no change in adverse outcomes [223]. A follow-up investigation [220] required participants to ingest up to 3.4 g/kg/day of protein for eight weeks while following a prescribed resistance training program and reported no changes in any of the blood parameters commonly used to assess clinical health (e.g., there was no effect on kidney or liver function). Their next study employed a crossover study design in twelve healthy resistance-trained men in which each participant was tested before and after for body composition as well as blood-markers of health and performance [221]. In one eight-week block, participants followed their normal (habitual) diet (2.6 g/kg/day) and in the other eight-week block, participants were prescribed to ingest greater than 3.0 g/kg/day resulting in an average protein intake of 2.9 g/kg/day over the entire 16-week study. No changes in body composition were reported, and importantly, no clinical side effects were observed throughout the study. Finally, the same group of authors published a one-year crossover study [222] in fourteen healthy resistance-trained men. When prescribed to a high protein diet, the participants were instructed to ingest 3 g/kg/day and achieved an average intake of 3.3 g/kg/day and when following their normal diet they consumed 2.5 g/kg/day. This investigation showed that the chronic consumption of a high protein diet (i.e., for 1 year) had no harmful effects on kidney or liver function. Furthermore, there were no alterations in clinical markers of metabolism and blood lipids.

### Key points

- Multiple review articles indicate that no controlled scientific evidence exists indicating that increased intakes of protein pose any health risks in healthy, exercising individuals.
- Statements by large regulatory bodies have also indicated that concerns about one’s health secondary to ingesting high amounts of protein are unfounded.
- A series of controlled investigations spanning up to one year in duration utilizing protein intakes of up to 2.5–3.3 g/kg/day in healthy resistance-trained individuals consistently indicate that increased intakes of protein exert no harmful effect on blood lipids or markers of kidney and liver function.

## Conclusion

In alignment with our previous position stand, it is the position of the International Society of Sports Nutrition that the majority of exercising individuals should consume at minimum approximately 1.4 to 2.0 g of protein per kg of bodyweight per day to optimize exercise training induced adaptations. Importantly, this recommendation also falls within the Institute of Medicine’s Acceptable Macronutrient Distribution Range (AMDR) of 10–35% protein [224]. The amount is dependent upon the mode and intensity of the exercise, the quality of the protein ingested, as well as the energy and carbohydrate status of the individual. However, it should be noted that there is preliminary evidence that consuming much higher quantities of protein (> 3 g/kg/d) may confer a benefit as it relates to body composition. Concerns that protein intake within this range is unhealthy are unfounded in healthy, exercising individuals. An attempt should be made to consume whole foods that contain high-quality (e.g., complete) sources of protein; however, supplemental protein is a safe and convenient method of ingesting high-quality dietary protein. The timing of protein intake in the period encompassing the exercise session may offer several benefits including improved recovery and greater gains in lean body mass. However, perhaps the most important issue regarding protein intake during the peri-workout period is that it serves as an opportunity to eat thus elevating one’s total daily protein intake. In addition, consuming protein pre-sleep has been shown to increase overnight MPS and next-morning metabolism acutely along with improvements in muscle size and strength over 12 weeks of resistance training. Intact protein supplements, EAAs and leucine have been shown to be beneficial for the exercising individual by increasing the rates of MPS, decreasing muscle protein degradation, and possibly aiding in recovery from exercise. In summary, increasing protein intake using whole foods as well as high-quality supplemental protein sources can improve the adaptive response to training.
